# Use of a Structured Mirrors Intervention Does Not Reduce Delirium Incidence But May Improve Factual Memory Encoding in Cardiac Surgical ICU Patients Aged Over 70 Years: A Pilot Time-Cluster Randomized Controlled Trial

**DOI:** 10.3389/fnagi.2016.00228

**Published:** 2016-09-28

**Authors:** Kimberly Giraud, Megan Pontin, Linda D. Sharples, Paul Fletcher, Tim Dalgleish, Allaina Eden, David P. Jenkins, Alain Vuylsteke

**Affiliations:** ^1^Cognitive Research Unit, Research & Development Department, Papworth Hospital NHS Foundation TrustCambridge, UK; ^2^Critical Care Unit, Papworth Hospital NHS Foundation TrustCambridge, UK; ^3^Leeds Institute of Clinical Trials Research, Faculty of Medicine and Health, University of LeedsLeeds, UK; ^4^Department of Psychiatry, School of Clinical Medicine, University of CambridgeCambridge, UK; ^5^Cognition, Emotion, and Mental Health Programme, Medical Research Council Cognition and Brain Sciences UnitCambridge, UK; ^6^Physiotherapy Department, Papworth Hospital NHS Foundation TrustCambridge, UK; ^7^Cardiac Services, Papworth Hospital NHS Foundation TrustCambridge, UK; ^8^Department of Cardiothoracic Anaesthesia & Intensive Care, Papworth Hospital NHS Foundation TrustCambridge, UK

**Keywords:** delirium, mirror, cardiac surgery, post-operative, factual memories, delusional memories, intensive care unit (ICU), post-traumatic stress disorder (PTSD)

## Abstract

**Introduction:** Post-operative delirium remains a significant problem, particularly in the older surgical patient. Previous evidence suggests that the provision of supplementary visual feedback about ones environment via the use of a mirror may positively impact on mental status and attention (core delirium diagnostic domains). We aimed to explore whether use of an evidence-based mirrors intervention could be effective in reducing delirium and improving post-operative outcomes such as factual memory encoding of the Intensive Care Unit (ICU) environment in older cardiac surgical patients.

**Methods:** This was a pilot time-cluster randomized controlled trial at a 32-bed ICU, enrolling 223 patients aged 70 years and over, admitted to ICU after elective or urgent cardiac surgery from October 29, 2012 to June 23, 2013. The Mirrors Group received a structured mirrors intervention at set times (e.g., following change in mental status). The Usual Care Group received the standard care without mirrors. Primary outcome was ICU delirium incidence; secondary outcomes were ICU delirium days, ICU days with altered mental status or inattention, total length of ICU stay, physical mobilization (balance confidence) at ICU discharge, recall of factual and delusional ICU memories at 12 weeks, Health-Related Quality of Life at 12 weeks, and acceptability of the intervention.

**Results:** The intervention was not associated with a significant reduction in ICU delirium incidence [Mirrors: 20/115 (17%); Usual Care: 17/108 (16%)] or duration [Mirrors: 1 (1–3); Usual Care: 2 (1–8)]. Use of the intervention on ICU was predictive of significantly higher recall of factual (but not delusional) items at 12 weeks after surgery (*p* = 0.003) and acceptability was high, with clinicians using mirrors at 86% of all recorded hourly observations. The intervention did not significantly impact on other secondary outcomes.

**Conclusion:** Use of a structured mirrors intervention on the post-operative ICU does not reduce delirium, but may result in improved factual memory encoding in older cardiac surgical patients. This effect may occur via mechanisms unrelated to delirium, altered mental status, or inattention. The intervention may provide a new means of improving outcomes in patients at risk of post-ICU anxiety and/or Post-Traumatic Stress Disorder.

**Trial Registration:** Clinicaltrials.gov identifier NCT01599689.

## Introduction

In spite of other improvements after cardiac surgery, post-operative delirium, an acute change in mental status and attention (Inouye, 2006), remains a significant problem (Rudolph et al., 2009). Currently affecting up to 50% of patients after cardiac surgery (Brown, 2014), delirium is independently associated with cognitive and functional decline 1 year later (Koster et al., 2009), increased mortality up to 10 years later (Gottesman et al., 2010) and significant healthcare and economic costs (Milbrandt et al., 2004). With advanced age constituting a major risk factor (National Institute for Heath, and Care Excellence [NICE], 2010), delirium will pose an increasing challenge to health care providers and policy makers as the older population increases. Pharmacological interventions have been shown effective in some studies with cardiac surgical and other patients (National Institute for Heath, and Care Excellence [NICE], 2010), but their prophylactic use is controversial (Page et al., 2013).

Current evidence-based guidelines suggest maintaining optimal sensory stimulation to reduce delirium in the post-operative ICU patient (National Institute for Heath, and Care Excellence [NICE], 2010). This may be particularly important in the case of older post-operative patients, who may suffer age-related sensory changes (Schneider et al., 2012; Hughes et al., 2015). We hypothesized that the provision of supplementary sensory feedback, via the use of mirrors, could help reduce post-operative delirium in older patients recovering after cardiac surgery. In previous studies with adult ICU patients in post-comatose states (Vanhaudenhuyse et al., 2008) and older care home residents with dementia (Tabak et al., 1996), the use of mirrors has been shown to positively impact on mental status and attention. Mental status and attention are core diagnostic domains for delirium (Inouye et al., 1990). In older patients recovering after stroke, the use of mirrors has been shown to support earlier physical mobilization (Altschuler et al., 1999; Sütbeyaz et al., 2007), which may further help reduce delirium risk in ICU patients (Schweickert et al., 2009).

A secondary and related hypothesis was that facilitating multisensory feedback and integration, via the use of mirrors, could support encoding of more factual or “real” events in the ICU environment, which may reduce development of “unreal” delusional memories after ICU discharge (Jones et al., 2001). Delusional memories, such as nightmares and hallucinations, are currently common after ICU discharge and have been associated with PTSD in this population (Jones et al., 2001, 2010).

Developing an evidence-based intervention on the basis of previous data (Tabak et al., 1996; Altschuler et al., 1999; Sütbeyaz et al., 2007; Vanhaudenhuyse et al., 2008; Freysteinson, 2009a,b), we devised a mirrors intervention which would support mental status and attention, earlier physical mobilization and recovery, and multisensory feedback and integration and which could be used in a post-operative ICU setting.

We aimed to explore whether this evidence-based intervention, used at set times such as following a change in mental status, during care-related procedures, or during routine physical therapy, could reduce delirium in older patients admitted to the ICU after cardiac surgery. We also aimed to explore whether use of this intervention positively impacts on post-operative outcomes such as factual (as opposed to delusional) memory encoding of the ICU environment. To achieve this, we used a cluster randomized controlled design with 2-week time period clusters of patients as unit of randomisation, in order to control for contamination.

## Methods

The study was approved by the National Research Ethics Service Committee East of England Cambridge Central (REC reference: 12/EE/0254, July 05, 2012). All participants gave written informed consent before taking part in any trial procedures. The trial was prospectively registered on ClinicalTrials.gov^1^.

The study population included patients aged 70 years and over, admitted to ICU after elective or urgent cardiac surgery over a 32-week period (October 29, 2012 to June 23, 2013). Exclusion criteria were: inability to obtain informed consent, care pathway anticipating admission elsewhere than to ICU following surgery, severe visual impairment impeding ability to recognize self in mirror, physical or communication barriers likely to impede effective administration of study procedures, severe mental disability likely to impede assessment of delirium, and history of psychiatric illness previously requiring hospitalization.

Consented patients were allocated to either the Mirrors Group or Usual Care Group at the time of their admission to ICU following cardiac surgery. Patients allocated to the Mirrors Group received a structured, protocol-driven mirrors intervention as part of their post-operative ICU care pathway. The intervention commenced from the time all anesthetic agents were switched off and the patient was awake following surgery. It was administered by patient’s nursing and physiotherapy teams and consisted in the use, and coaching in the use, of two types of mirrors to support mental status and attention, physical mobilization, and multisensory feedback and integration. The mirrors included: (i) a standard 23 × 41 cm unbreakable personal mirror of the type used in clinical/therapeutic settings where viewing of the face is desired (e.g., speech therapy) and (ii) a standard 160 × 50 cm mobile posture mirror of the type used in physical/occupational therapy to provide visual feedback supporting proprioception (e.g., rehabilitation following stroke). A protocol determined the times of use and standardized ways in which clinicians should use these mirrors (see **Table 1**).

**Table 1:** Structured Mirrors protocol, defining indicated times and instructions for use of mirrors.

Indicated times of use	Therapeutic action/Instructions for use
(1) Patient shows change in mental status (i.e., on sedation scale) or wakes from natural sleep	*To support mental status and attention:* Patient coached in use of mirror as reorienting tool and for supporting self-awareness

(2) Patient to have medical/nursing procedures administered to them (e.g., dressing change, line removal)	*To support multisensory feedback and integration:* Mirror used to aid explanation about procedures to patient and awareness of objects/events in patients personal space, patient coached in use of mirror as communication tool for asking about what is happening to/going on around them

(3) Patient needs help with personal care, grooming, eating, etc. (4) Patient to have passive and active limb exercises with ICU physiotherapist (5) Patient to have routine mobilization exercises with ICU physiotherapist (e.g., sitting on edge of bed, bed-to-chair transfers)	*To support physical mobilization:* Mirror placed in optimal safe position for viewing. Patient coached in using mirror to obtain visual feedback to support hand-eye coordination and allow self-care. Patient coached in using visual feedback to understand body and limb positions, monitor limb trajectories, trunk control, posture and balance, and promote earlier mobilization.

Patients allocated to the Usual Care Group received the current standard post-surgical ICU care which includes no prescriptions around the use of mirrors. If a Usual Care patient brought in a mirror with their personal belongings, they were allowed to use as it in the way that they wished, as would occur in routine practice. Both groups received the same 1:1 intensive care and continuous presence of a nurse at the bedside. Clinical care of study patients (including the management of delirium) was in no way affected by group allocation or participation in the study.

The *primary endpoint* was delirium incidence, defined as the proportion of patients with at least one recorded episode of delirium during their ICU stay. Delirium was diagnosed using the Confusion Assessment Method for the ICU (CAM-ICU) (Ely et al., 2001a,b), administered to all patients (except if deeply sedated or comatose) by patients’ direct care teams, twice per day, according to the CAM-ICU Manual (Vanderbilt University Medical Center, 2014) and recommended clinical guidelines (National Institute for Heath, and Care Excellence [NICE], 2010). The ICC for delirium incidence was also calculated to quantify similarity of outcomes within clusters. *Secondary endpoints* were: ICU days with delirium, proportion of ICU LOS with delirium, ICU days with altered mental status and inattention as assessed by direct care teams using the RASS (Sessler et al., 2002) and CAM-ICU, physical mobilization at ICU discharge as assessed by a research nurse using the ABC Scale (Powell and Myers, 1995), total length of ICU stay, factual memories and incidence of delusional memories at 12 weeks after surgery as assessed by a research nurse using the ICUMT (Jones et al., 2000), HRQoL at 12 weeks after surgery assessed by a research nurse using the EQ-5D VAS and index score (EuroQol Group, 1990), and acceptability of the intervention assessed on the basis of the proportion of mirror uses as indicated, proportion of instances where patients refused, and total adherence rate.

The unit of randomisation was 2-week time period cluster (with all consented patients admitted to ICU during a given 2-week time period cluster being allocated to the treatment of that cluster), in order to reduce the risk of contamination associated with the visible nature of the intervention in an open-ICU environment. There were 16 2-week clusters in total (eight Mirrors, eight Usual Care). Each cluster included a 2-day washout period at the end, during which admitted patients were excluded from being enrolled (this duration was considered optimal on the basis of our ICU admission statistics for ensuring that >90% of enrolled patients would be discharged before the end of their cluster). The treatment for each cluster was determined by the trial statistician (LDS) using a computer-generated random permutation algorithm ensuring equal numbers of clusters assigned to each treatment arm [Stata version 12 (StataCorp, 2011)]. Allocation was revealed to the clinical trial coordinator on the morning of the start of each cluster using an independent telephone randomisation system. Treatment allocation was the same for all patients admitted within a same cluster and continued until patients’ ICU discharge, even if their ICU stay extended into a subsequent cluster with different treatment allocation. Blinding of patients and clinicians to group allocation was not possible due to the nature of the intervention. To minimize subjective bias and other effects, an objective and standardized screening methodology was followed to measure delirium outcomes and members of the research team carrying out data analysis were blinded to treatment allocation.

No formal sample size calculation was carried out in this pilot study. Instead, a pragmatic sample of patients receiving cardiac surgery over a 32-week period was recruited in order to produce preliminary estimates of the incidence of delirium in the two groups and provide an estimate of the ICC from which to estimate sample size for a subsequent definitive trial (if warranted). The effects of the intervention on outcome measures were analyzed according to the intention-to-treat principle. Delirium incidence and incidence of delusional memories were analyzed using mixed effects logistic regression with random intercepts for the time clusters and assuming an exchangeable covariance structure. Days with delirium, days with altered mental status and inattention, length of ICU stay, and number of factual memories were analyzed using mixed effects Poisson regression using a modified approach with robust error variances (Zou, 2004) to estimate the relative risk and confidence intervals. Proportion of ICU stay with delirium, physical mobilization (ABC scores), HRQoL (VAS and index scores) were analyzed using a generalized linear model with a logit link and binomial family, using the robust option to obtain standard errors (Papke and Wooldridge, 1996). All mixed model analyses were carried out adjusting for patient characteristics [age, gender, and operative risk (EuroSCORE, Nashef et al., 1999)] (included as fixed effects) and time-clusters (included as a random effect). All analyses were carried out using Stata version 12 (StataCorp, 2011).

## Results

**FIGURE 1:**
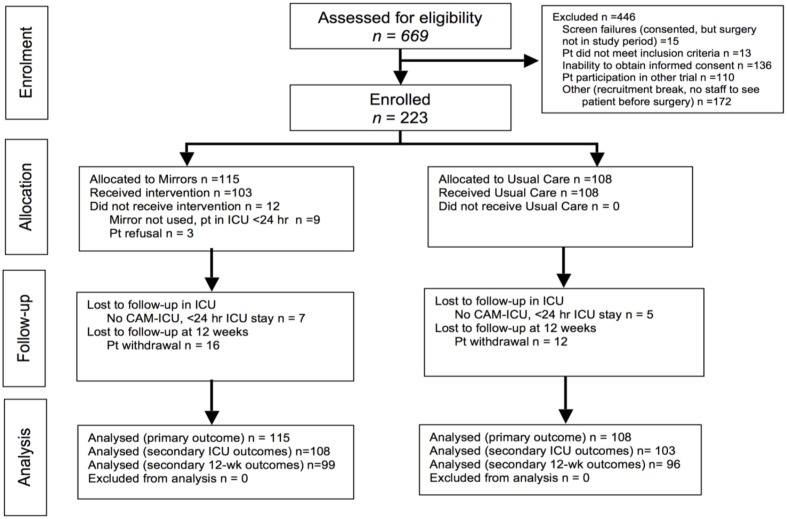
**CONSORT diagram showing flow of participants through the trial**.

Over the 32-week period between October 29, 2012 to June 23, 2013, eight clusters were assigned to Mirrors (*n* = 115) and eight clusters to Usual Care (*n* = 108). Among 669 patients screened, 223 were enrolled onto the study and 446 were excluded (see **Figure 1** for details). The study population had a mean age of 77.2 (SD:4.9), was mostly male, and had a mean logistic EuroSCORE of 6.7 (IQR: 3.8–13.0). The cluster randomisation was successful in creating groups of equal age, gender, surgical risk, surgery type, and priority (see **Table 2**).

**Table 2:** Patient background and operative characteristics.

	Mirrors group (*n* = 115)	Usual care group (*n* = 108)	Total (*N* = 223)
Age	77.4 (4.8)	77.0 (4.9)	77.2 (4.9)
Gender	28 (24%)	25 (23%)	53 (24%)
EuroSCORE	7 (5–9 [3–16])	7 (5–9 [3–18])	7 (5–7 [3–18])
(additive; logistic)	6.8 (3.7–13.0 [1.8–58.6])	6.7 (3.8–13.2 [1.8–80.8])	6.7 (3.8–13.0) [1.8–80.8])
**Surgery Type:**			
–CABG	58 (50%)	54 (50%)	112 (50%)
–AVR	17 (15%)	12 (11%)	29 (13%)
–MVR	9 (8%)	9 (8%)	18 (8%)
–CABG+AVR	16 (14%)	17 (16%)	33 (15%)
–CABG+MVR	2 (2%)	2 (2%)	4 (2%)
–AVR+MVR	4 (3%)	4 (4%)	8 (4%)
–Other complex	9 (8%)	10 (9%)	19 (9%)
**Priority:**			
–Elective	82 (71%)	75 (69%)	157 (70%)
–Urgent	33 (29%)	33 (31%)	66 (30%)

*Values are mean (SD), median (IQR [range]), or number (proportion)*.

*CABG, coronary artery bypass graft; AVR, aortic valve replacement; MVR, mitral valve replacement*.

### Effects of the Intervention

**Table 3** summarizes results for primary outcome (delirium incidence), as well as other delirium and non-delirium outcomes. There were no significant differences between groups in delirium incidence. There were no significant differences in median days with delirium or proportion of total ICU stay with delirium. The ICC was <0.01 (not shown), indicating that time clustering effects were negligible. Mirrors were independently associated with greater recall of factual items from ICU at 12 weeks, with the rate of recall for Mirrors patients, relative to the Usual Care Group, being 1.34, 95% CI 1.10 to 1.62, *p* = 0.003 (significant at Bonferroni-adjusted alpha of 0.005)]. This positive effect was observed in both male and female patients, irrespective of age and operative risk [even though older age was itself a predictor of poorer factual recall (rate of recall for patients aged over 80 years, relative to patients aged 70–80 years: 0.74, 95% CI 0.59 to 0.93, *p* < 0.001, not shown in table)]. There were no other significant differences in secondary outcomes between groups.

**Table 3:** Comparison of delirium and other outcomes between Mirrors and Usual Care groups.

Delirium ICU outcomes	Mirrors *(n = 115)*	Usual Care *(n = 108)*	Estimate	95% CIs	*p*-value
Number (incidence) of patients with Delirium	20 (17%)	17 (16%)	OR 1.15	0.54 to 2.43	0.705
Delirium duration; days	1 (1–3 [1–25])	2 (1–8 [1–13])	RR 0.66	0.25 to 1.75	0.401^∗^
			RR 0.66	0.26 to 1.70	0.393^†^
Delirium duration; proportion of ICU stay	0.54 (0.30)	0.65 (0.29)	Coef -0.17	-0.56 to 0.23	0.406^∗^
			Coef -0.10	-0.67 to 0.47	0.729^†^

**Non-Delirium ICU outcomes**	**Mirrors *(n = 108)***	**Usual Care *(n = 103)***	**Estimate**	**95% CIs**	***p*-value**

Altered mental status; days	1 (1–2 [1–33])	1 (1–2 [1–22])	RR 0.58	0.29 to 1.16	0.123^∗^
			RR 0.58	0.30 to 1.12	0.103^†^
Inattention; days	1 (1–2 [1–32])	1 (1–2 [1–18])	RR 0.65	0.25 to 1.68	0.374^∗^
			RR 0.70	0.24 to 1.86	0.441^†^
ABC Score‡	0.26 (0.23)	0.20 (0.16)	Coef 0.27	-0.10 to 0.64	0.147
Total ICU stay; days	2 (1–2 [1–55])	2 (12 [1–38])	RR 0.99	0.75 to 1.31	0.942

**Non-Delirium 12-week outcomes**	**Mirrors *(n = 99)***	**Usual care *(n = 96)***	**Estimate**	**95% CIs**	***p*-value**

Factual memories; items recalled	4 (2–6 [0–8])	3 (1–5 [0–8])	RR 1.34	1.10 to 1.62	0.003
Number (incidence) of patients with delusional memories	26 (26%)	23 (24%)	OR 1.22	0.63 to 2.36	0.546
EQ-5D VAS^§^	73 (19)	77 (15)	Coef -0.04	-0.09 to 0.01	0.127
EQ-5D index	0.87 (0.13)	0.87 (0.13)	Coef -0.00	-0.05 to 0.04	0.950

*Values are mean (SD), median (IQR [range]), or number (proportion). Model estimates, 95% Confidence intervals (CIs), and *p*-values are also shown*.

*^∗^In patients with at least one occurrence of Delirium, altered Mental Status, or Inattention (whichever is being measured)*.

*†In total sample with data*.

*‡Activities-specific balance confidence (ABC) Score*.

*^§^EQ-5D Visual Analog Score*.

**Table 4** shows total mirror uses (by type), as well as non-uses, out of all recorded nursing and physiotherapy observations of patients in the Mirrors Group. Mirrors were used by ICU nurses and physiotherapists and accepted by patients in 86% of all observations. The number of Mirrors patients who refused the intervention altogether was low (3%, not shown). “Support for medical/nursing procedures” represented the most frequent type of mirror use.

**Table 4:** Number and rate of mirror uses (and non-uses) out of total recorded observations.

	Total recorded observations (*N* = 1880)
Change in mental status	303 (16%)
Medical/nursing procedures	450 (24%)
Personal care/grooming	378 (20%)
Passive/active limb exercises	180 (10%)
Mobilization exercises	166 (9%)
Other^∗^	139 (7%)
Non-uses due to patient refusal^†^	264 (14%)
Total uses as indicated	1616 (86%)

*^∗^Examples of other uses recorded by clinicians were: to support deep breathing exercises, for visualising chest drains to contextualise pain, for passive viewing of self as per patient request*.

*†Including in patients who never received intervention*.

## Discussion

Results of this study do not support the hypothesis that use of this structured mirrors intervention on ICU reduces post-operative delirium in cardiac surgical patients. Evidence for effective non-pharmacological interventions in cardiac surgical ICU patients is lacking (Brown, 2014). In the current pilot study, we recruited a pragmatic sample of cardiac surgical patients aged 70 years and over (with and without delirium risk factors) over 32 weeks at a single center. Given that most patients in our sample were discharged from ICU less than 2 days after surgery and neither study treatments nor delirium testing were carried out beyond ICU discharge, it is possible that treatment effects (and post-operative delirium rates) were under-estimated in the present study.

The use of mirrors on ICU was associated with a small, but statistically significant positive impact on recall of factual (as opposed to delusional) items from ICU at 12 weeks after surgery, even after adjusting for multiple comparisons. This effect was observed in both male and female patients, irrespective of patient characteristics and operative risk. Long-term anxiety and PTSD currently represent a significant problem in surgical, as well as non-surgical, ICU patients (Jones et al., 2001, 2010; Girard et al., 2007). A substantial body of evidence suggests that the number of factual memories (as opposed to delusion memories) after ICU discharge is a predictor of subsequent post-ICU PTSD (Jones et al., 2001). Indeed, numerous strategies supporting factual encoding [e.g., the use of patient diaries (Jones et al., 2010), sedation reduction (Sackey et al., 2008)] have been investigated to improve long-term outcomes in ICU patients. On the basis of evidence from other settings, we had hypothesized that use of mirrors would positively impact on mental status and attention, thereby enhancing factual encoding. However, our results do not support this mechanism as quantified on the basis of CAM-ICU feature data. It is possible that use of mirrors during patients’ post-operative ICU care can help form a more integrated and predictable percept of self in an unexpected environment to normalize the balance between feed-forward and feedback signaling (Corlett et al., 2007; Murray et al., 2010) – a disruption of which older patients (aged >80 years) may be at increased risk. Further exploration of the potential of mirrors to help offset this risk and improve psychiatric outcomes in a larger sample of older cardiac surgical (and other) patients at increased risk may be warranted.

Some methodological considerations of this study should be noted. First, we are unable to rule out placebo and other effects. It is unlikely that use of mirrors was associated with ‘additional’ care procedures administered to patients in the Mirrors Group, as all patients in both groups received continuous 1:1 ICU nursing at the bedside and the intervention was designed for use during aspects of care that were already routinely administered. Use of mirrors may have been associated with increased staff-patient interaction to patients in the Mirrors Group (Freysteinson, 2009b), but staff-patient interaction is prerequisite to helping them reorient and, indeed, the potential of mirrors to support patient interaction was integral to our hypothesis. Second, while the cluster randomisation was successful in creating groups of equal age, gender, operative risk, surgery type, and surgical priority, we cannot rule out a contribution of pre-existing cognitive differences to between-group results, as baseline cognitive testing was not carried out.

While not supporting effectiveness in reducing delirium, these pilot results suggest that use of a structured mirrors intervention as part of older cardiac surgical patients’ post-operative ICU care could result in improved recall of factual (but not delusional) ICU memories 12 weeks later. The intervention could provide a simple new means of improving outcomes in patients at risk of post-ICU anxiety and/or PTSD.

## Author Contributions

KG designed and coordinated the study, collected the data, participated in data analysis, and drafted the manuscript; MP helped conceive of the study, design the intervention, and draft the manuscript; LS helped design the study, carried out data analysis, and critically revised the manuscript; PF helped design outcome measures, interpret the results, and draft the manuscript; TD helped design the study and draft the manuscript; AE helped design the intervention and outcome measures and draft the manuscript; AV helped design the study, supervised the conduct of the study, and helped draft and critically revised the manuscript. All authors read and approved the manuscript.

## Conflict of Interest Statement

The authors declare that the research was conducted in the absence of any commercial or financial relationships that could be construed as a potential conflict of interest.

## Footnotes

1 http://www.clinicaltrials.gov/ct2/show/NCT01599689

## Abbreviations

ABC: activities-specific balance confidence
AVR: aortic valve replacement
CABG: coronary artery bypass graft
CAM-ICU: confusion assessment method for the intensive care unit
EQ-5D: European quality of life-5 dimensions
EuroSCORE: European system for cardiac operative risk evaluation
HRQoL: health-related quality of life
ICC: intra-cluster correlation coefficient
ICU: intensive care unit
ICUMT: intensive care unit memory tool
LOS: length of stay
MVR: mitral valve replacement
PTSD: post-traumatic stress disorder
RASS: Richmond agitation-sedation scale
REC: research ethics committee
VAS: visual analog scale
